# The Feasibility, Acceptability, and Efficacy of Delivering Internet-Based Self-Help and Guided Self-Help Interventions for Generalized Anxiety Disorder to Indian University Students: Design of a Randomized Controlled Trial

**DOI:** 10.2196/resprot.4783

**Published:** 2015-12-11

**Authors:** Nitya Kanuri, Michelle G Newman, Josef I Ruzek, Eric Kuhn, M Manjula, Megan Jones, Neil Thomas, Jo-Anne M Abbott, Smita Sharma, C. Barr Taylor

**Affiliations:** ^1^ Behavioral Medicine Lab Department of Psychiatry and Behavioral Sciences Stanford University School of Medicine Stanford, CA United States; ^2^ mHealth Institute Palo Alto University Palo Alto, CA United States; ^3^ Department of Psychology and Psychiatry The Pennsylvania State University University Park, PA United States; ^4^ Dissemination and Training Division National Center for PTSD VA Palo Alto Health Care System Menlo Park, CA United States; ^5^ Department of Psychiatry and Behavioral Sciences Stanford University School of Medicine Stanford, CA United States; ^6^ Department of Clinical Psychology National Institute of Mental Health & Neuro Sciences Bengaluru India; ^7^ Lantern San Francisco, CA United States; ^8^ Brain and Psychological Sciences Research Centre Swinburne University of Technology Hawthorn Australia; ^9^ Reach Beyond NGO, India Hyderabad India; ^10^ Academic Registration & Counseling Division Birla Institute of Technology & Science, Pilani, Hyderabad Campus Hyderabad India

**Keywords:** Internet-delivered cognitive behavioral therapy (iCBT), generalized anxiety disorder, college health screening, low- and middle-income countries, randomized controlled trial, guided self-help, quality of life

## Abstract

**Background:**

Generalized anxiety disorder (GAD) is one of the most common mental disorders among university students; however, many students go untreated due to treatment costs, stigma concerns, and limited access to trained mental health professionals. These barriers are heightened in universities in India, where there are scant mental health care services and severe stigma surrounding help seeking.

**Objective:**

To evaluate the feasibility, acceptability, and efficacy of Internet-based, or “online,” cognitive behavioral therapy (CBT)-based unguided and guided self-help interventions (using the programs GAD Online and Lantern, respectively) to reduce GAD symptoms in students with clinical and subthreshold GAD and, ultimately, reduce the prevalence and incidence of GAD among the student population.

**Methods:**

Students will be recruited via 3 colleges in Hyderabad, India, and referred for a campus-wide online screening. Self-report data will be collected entirely online. A total of 300 qualifying students will be randomized in a 1:1:1 ratio to receive GAD Online, Lantern, or to be in a wait-list control condition, stratified by clinical and subthreshold GAD symptomatology. Students will complete a postintervention assessment after 3 months and a follow-up assessment 6 months later, at which point students in the wait-list control condition will receive one of the programs. The primary outcome is GAD symptom severity at 3 months postintervention. Secondary outcomes include GAD caseness at 9 months, other anxiety and depression symptoms, self-efficacy, and functional measures (eg, sleep, social functioning) at 3 and 9 months, respectively. Primary analyses will be differences between each of the intervention groups and the wait-list control group, analyzed on an intention-to-treat (ITT) basis using mixed-design ANOVA.

**Results:**

The study commenced in February 2015. The sample was recruited over a 3-week period at each college. The trial is expected to end in December 2015.

**Conclusions:**

This trial will be the first to evaluate the use of Internet-based CBT programs compared with a wait-list control group for the treatment of GAD among students in Indian universities. If effective, these programs have the potential to reduce the mental health care treatment gap by providing readily accessible, private, and cost-effective evidence-based care to students with GAD who do not currently receive the treatment they need.

**Trial Registration:**

ClinicalTrials.gov NCT02410265 http://clinicaltrials.gov/ct2/show/NCT02410265 (Archived by WebCite at http://www.webcitation.org/6ddqH6Rbt).

## Introduction

### Background

Anxiety disorders are the most prevalent class of mental health disorders across the world, with an estimated lifetime prevalence of 4.8-31.0% [1]. Generalized anxiety disorder (GAD), in particular, is one of the most common disorders. In a nationally representative sample in the United States, there was a 5.7% prevalence of GAD [2], and in 14,175 students across 26 United States college campuses, there was a 7% prevalence of GAD [3]. Comparably, in India, a meta-analysis of 13 psychiatric epidemiological studies (N=33,572) conducted in urban and rural India across all age groups yielded an estimated prevalence rate of 5.8% for GAD [4]. Similarly, Nair et al [5] found a 6.6% prevalence of GAD among adolescents in India. In Indian universities, in particular, Sahoo and Khess [6] found a 19% prevalence of GAD in 405 young male university students. These studies suggest that anxiety is a major public health concern, particularly in Indian students. Given that the global average age of onset of GAD is in adolescence and in early adulthood [1,5,7,8], university students are a vulnerable population.

If left untreated, GAD has been demonstrated to have a chronic course and persistent symptoms [9] and is associated with significant distress, disability, quality of life, and medical problems [10]. In addition, subthreshold GAD cases (ie, individuals with significant symptoms who do not meet the full diagnostic criteria for GAD) have also proven equally costly in terms of functional impairment, medically unexplained symptoms such as pain [11], quality of life [12], disability, and help seeking [13,14]. The presence of GAD symptoms even increases the cost of health care from twofold to greater than fourfold [15], with disorder severity positively correlated with total medical costs [16]. Furthermore, both GAD and subthreshold GAD are significant predictors of first onset of other anxiety, mood, substance-use, and impulse-control disorders [17,18]. Studies that have evaluated possible GAD risk factors suggest that subthreshold symptoms might predict onset of GAD [18-20]. Thus, reducing GAD symptomatology among young people and targeting both treatment and prevention have tremendous public-health significance, not least of which is the potential to mitigate ongoing disability and costs [21].

Unfortunately, the majority of affected young people do not receive treatment. Young et al [22] estimated that only 20% of young people in the United States receive adequate treatment. In university environments, students often do not seek treatment due to barriers such as time, stigma concerns, treatment cost, insufficient information about their disorder or available treatment [23,24], and particularly in India, confidentiality concerns [25]. Hunt and Eisenberg [23] discovered that students indicate a preference for self-management and the perception that issues are not serious enough to warrant treatment, and both these are considered primary reasons for not seeking help.

Such stigma around mental health may be a larger issue in Indian versus US populations. Among Asian cultures, research has documented a belief that “emotional reactions” do not merit professional intervention [26]. Another study among Indian adolescents documented a perception that having a mental illness is shameful [27]. Stigma may result in resistance to seeking treatment and those who might seek treatment may be reluctant for fear of discrimination. Online programs offer the advantage of reducing some aspects of stigma.

Even more impactful than the issue of low help seeking is a lack of treatment availability. In the United States, inadequate counselor availability is a significant issue [23]; in developing countries, the issue is significantly worse. In India, for example, there are only 5000 licensed mental health professionals; in the United States, there are 550,000 to treat a population one-fourth of India’s size [28]. This equates to roughly 1 professional per 580 individuals in the United States and 1 per 250,000 in India. There is, therefore, considerable opportunity to improve mental health treatment for students in India by increasing access to treatments that do not require mental health professionals to deliver them.

Internet-based, or “online,” self-help interventions have the potential to overcome barriers such as stigma, cost, and limited specialist services. Online, unguided and guided self-help interventions offer an opportunity to provide cost-effective, evidence-based care to a large population simultaneously [29]. Unguided, purely self-help (SH) interventions have proven efficacious for treating individuals diagnosed with anxiety disorders including GAD [30-36]. In one study, individuals identified as having clinical GAD received a fully automated SH program and achieved significant improvement across primary symptom severity measures as well as secondary measures such as self-confidence in managing mental health issues and quality of life [32]. Guided self-help (GSH) interventions, in which an online program guide or “coach” [37] supports and guides a user by monitoring progress in the program and providing personalized feedback and encouragement typically via messaging and/or phone, have proven even more effective [38,39]. In fact, GSH interventions have been demonstrated to be as effective as in-person therapy for treating clinical anxiety disorders [39] and depression [40]. These findings suggest that less costly, Internet-based SH and GSH interventions can be considered adequate alternatives to traditional in-person therapy. However, there is no research examining the efficacy of these interventions to reduce GAD symptomatology in students in Indian universities who currently have limited access to mental health care. Therefore, this study seeks to evaluate the feasibility, acceptability, and efficacy of Internet-based interventions, both unguided (using the GAD Online program) and guided (using the Lantern program), to reduce GAD symptoms in Indian university students.

### Objectives and Hypotheses

The objective of this trial is to evaluate the efficacy of the GAD Online and Lantern programs for Indian university students with clinical or subthreshold GAD. The primary hypothesis is that the use of either of the active intervention groups, the unguided GAD Online program or the guided Lantern program, for 3 months will lead to greater GAD symptom reduction than will be seen among students in the wait-list control group. The secondary hypothesis is that a guided intervention (Lantern) will lead to greater user engagement and symptom reduction than the unguided intervention (GAD Online). In addition, it is hypothesized that the use of either program will lead to greater functional gains (eg, social functioning) and improvements in mental health self-efficacy than will be seen among students in the wait-list control condition. Table 1 provides a summary of the trial outcome measures.

## Methods

### Stakeholder Engagement

To better understand the cultural context, existing mental health care system, and needs, a community-based participatory research (CBPR) methodology was used [41]. CBPR calls for a collaborative approach to research that involves all stakeholders in the research process to ensure findings and knowledge gained are meaningful to the community and recommendations are feasible to implement and sustainable. A series of interviews and co-learning discussions were conducted with the following key stakeholders: university administration, existing on-campus counselors and medical professionals, faculty mentors, hostel wardens, and student groups. In interviews with university administration, key topics addressed included current student welfare priorities and where mental health care falls on the list, successful and failed student outreach and engagement efforts, perspectives and concerns of parents, and potential budget for implementing and sustaining new mental health care resources. On-campus counselors provided insight into the typical structure of counseling in Indian school and university environments and the relationship between administration and provider, particularly highlighting that ethics around patient/student privacy are not as widely or consistently practiced in India. Faculty provided the perspective of a “mentor” who primarily gets assigned students flagged for poor academic performance. Hostel wardens provided insight into how “trouble cases” are identified and triaged. Finally, students shared information on student knowledge of resources, barriers that exist to accessing them, and the general campus culture around mental health, particularly noting the stigma around anything involving the term “mental.” The details and results of these qualitative assessments will be written up separately.

### Preliminary Feasibility and Acceptability Evaluation

To evaluate the feasibility of disseminating these programs to university populations in India, interviews were conducted with university administration to assess the ability to conduct outreach initiatives to engage and educate students about these programs and circulate a campus-wide online survey to assess students’ mental health and connect those with significant symptoms to online interventions. Administrators were interested in the proposed survey-linked-to-intervention approach, specifically noting students’ interest in opportunities that might allow them to access help privately and on their own time; however, they expressed hesitation about whether students would actually use online programs for mental health care.

To evaluate the acceptability of these Internet-based mental health care interventions among the Indian university student population, a presentation and survey was conducted in April 2014 at one Indian university. Following a presentation to an auditorium of nearly 300 students about anxiety and online programs designed to reduce associated symptoms, students were invited to the adjacent computer laboratory to complete an online survey assessing anxiety symptom severity and student interest in using these types of online programs. The survey was completed by 78 undergraduate students (aged over 18; 52% male). Of these, 94% (73/78) indicated they would consider using “a mobile phone app-based “coached” program” to help them with anxiety symptoms (the Lantern Internet program is mobile optimized so users accessing the website via their mobile phones can have a similar experience to those able to use the iPhone iOS mobile app; therefore, the language “app based” was used). The majority (55/78, 70%) indicated they would like to be contacted should these programs become available at their college.

Of the students who completed the survey, 10% (8/78) had clinical GAD as measured by the 5th Edition of the Diagnostic and Statistical Manual of Mental Disorders (DSM-5) criterion-based scoring of the GAD-Q-IV [42], and an additional 22% (17/78) had subthreshold GAD, defined as a score of 5.7 or above on the GAD-Q-IV but not being clinical. Additionally, 24% (19/78) had clinical social anxiety as measured by DSM-V criterion-based scoring of the Social Phobia Diagnostic Questionnaire (SPDQ) [43], 13% (10/78) scored above a clinical cutoff of 8.75 on the Panic Disorder Self Report (PDSR) [44], and 8% (6/78) scored above a clinical cutoff of 38 on the PTSD Checklist for DSM-V (PCL-5) [45]. The prevalence is roughly comparable with that reported by Raakhee and Aparna [46] in a population (N=100) of higher secondary students in India: 13% GAD, 15.6% social anxiety, 15% panic disorder, with 56.8% of all students experiencing 1 or more types of anxiety disorders. Similarly, the prevalence of GAD is comparable with the 19% found by Sahoo and Khess [6] via clinical interviews in a population (N=405) of young adult males in an Indian university. This informal assessment bolstered the belief that the Indian university student population is interested in and might benefit from access to Internet-based interventions addressing anxiety.

### Setting

The trial will be conducted in 3 private colleges in Hyderabad, Telangana, a city in the south of India.

### Design

A parallel arm, 3 (condition) × 3 (time) randomized controlled trial with equal allocation of students between arms will be used. Students will be randomized to the unguided GAD Online intervention, the guided Lantern intervention, or a wait-list control group. Those assigned to the GAD Online or Lantern group will have full program access for 3 months. Outcomes will be assessed at screening/baseline, postintervention (3 months), and 6-month follow-up (9 months).

### Participants and Procedures

#### Recruitment

**Figure 1:**
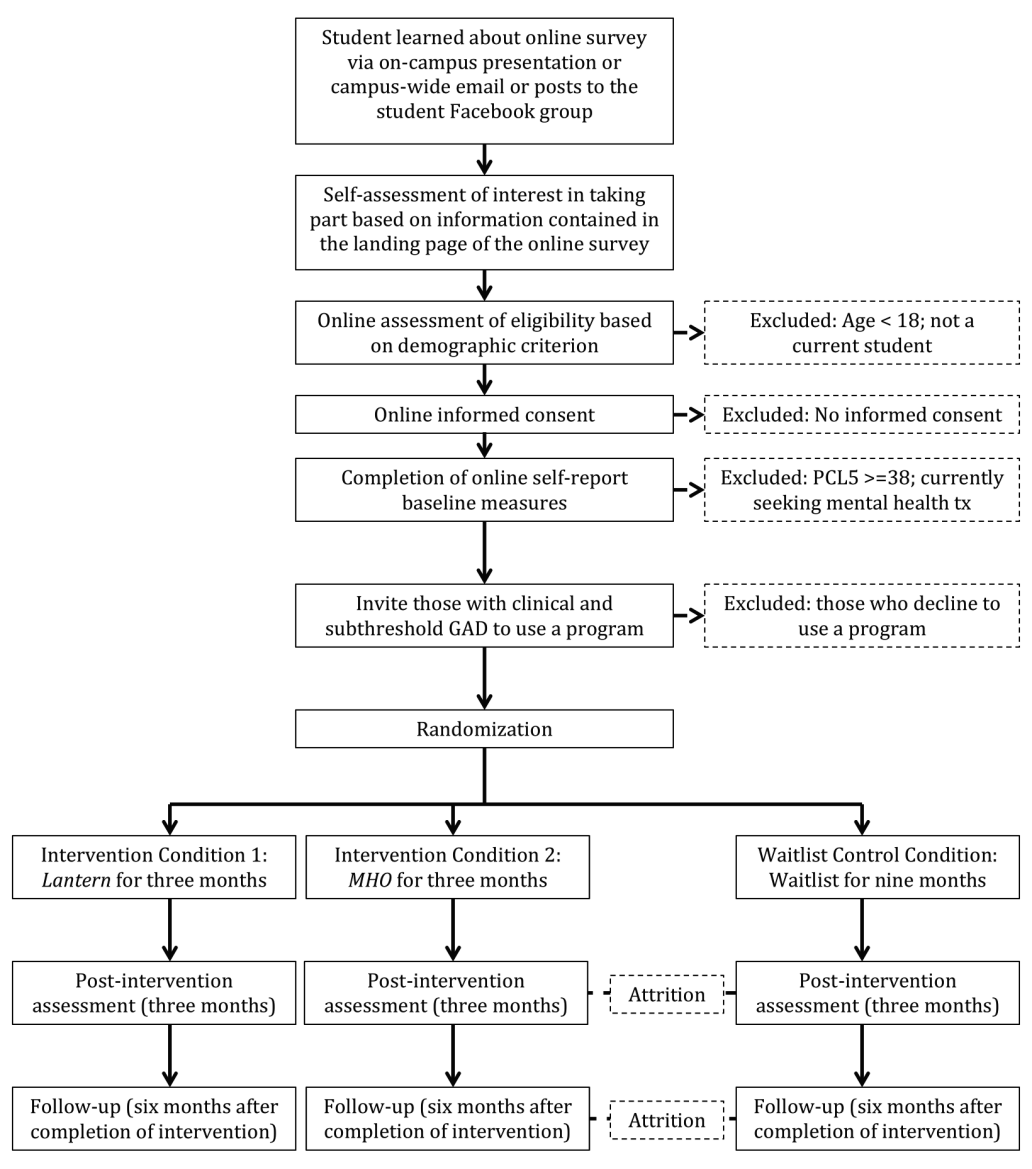
Trial flow chart.

The flowchart (Figure 1) shows the process of recruitment and follow-up of students in the trial. Potential participants will be recruited through an online survey delivered across 3 colleges in Hyderabad. The research coordinator will visit each site and deliver presentations about the study as well as send campus-wide emails. After raising awareness about the study across the campus, the research coordinator will circulate a link to an online survey via email, which will also be posted in the student Facebook page and other social media groups. The landing page will again provide information about the study and instruct interested individuals to move forward to complete an online consent form and screening survey. This landing page will also contain the contact information of the on-campus counselor, as this information is meant to be available to all students across campus.

Those who meet criteria for clinical or subthreshold GAD will be invited to participate in the study and use an online program. Those who are interested will be informed that they will be assigned to a condition within 4 weeks. Everyone will receive their assignment email within 4 weeks following baseline, and those assigned into the 2 treatment conditions may activate their program account and begin immediately. Students in the GAD Online or Lantern conditions will have access to the intervention over a 3-month period. Students assigned to GAD Online can access content at whatever pace they choose. Students assigned to Lantern can access up to1 session per day because the program is structured to build mastery. Program access will be disconnected following this period. If students still meet clinical criteria for GAD at the end of the intervention, they will receive a referral to visit the on-campus counselor. Students will also be given a list of free, publicly available online self-help resources for anxiety (eg, Mental Health Online, ThisWayUp). Continuation in the guided intervention will not be possible due to resource constraints around program coach availability in the context of this research trial. Assessments will be conducted completely online. At each assessment time point, students will receive an email with a link to an online survey hosted on Qualtrics, an online survey platform with industry-standard data security measures.

#### Eligibility

Eligible participants will be current students of the colleges, all of which are English medium, aged 18 or older who provide their email address, informed consent to complete the study, and meet criteria for clinical or subthreshold GAD. This age range was chosen because individuals younger than 18 require parental consent for participation in research. Based on self-report responses in the online survey, students are categorized into clinical, subthreshold, and asymptomatic for GAD. Those who meet DSM-V diagnostic criteria for GAD as measured by criterion-based scoring of the 4th edition of the Generalized Anxiety Disorder Questionnaire (GAD-Q-IV) [42] are classified as clinical, or more realistically,

“a probable diagnosis of GAD based on self-report.” To note, the diagnostic criteria did not change from DSM-IV to DSM-V, and therefore the GAD-Q-IV questions can still be used to assess DSM-V GAD criteria. Those who score 5.7 or above using dimensional scoring of the GAD-Q-IV but are not clinical are classified as “subthreshold.” All others are asymptomatic. It is also important to note that clinical diagnosis should be established using a clinical interview, which is not feasible in this study, thus the use of the self-report measures. Moreover, this study’s aim is not to provide diagnoses to students but rather to use gold-standard instruments to provide appropriate “matches” between students and available resources (ie, the research study or referral).

The PTSD Checklist for DSM-V (PCL-5) [45] will be used to refer individuals who self-report clinical symptoms of PTSD to targeted intervention or clinical services. CBT for GAD has been demonstrated to improve symptoms across comorbid disorders such as social anxiety disorder and depression [47], and therefore a “pure GAD” sample is not necessary. However, PTSD requires a more specific approach, and data on CBT for GAD’s impact on PTSD are not available. A more conservative approach was followed: PTSD “probability” based on the PCL-5 (ie, scoring 38 or above) will result in a referral to seek help from the on-campus counselor and to access the free, publicly available iPhone and Android mobile app, PTSD Coach [48]. It is difficult to get a “clean sample,” so a balance between safety and generalizability was pursued. No additional symptom-based exclusion criteria were imposed, because a primary goal of this study was to connect most students to a service they might be willing to access. Once a student is connected to a program, mechanisms exist to ensure referrals are given if symptoms are not adequately reduced and/or other symptoms present themselves (eg, program coaches give referrals to those in the guided Lantern intervention and postintervention survey feedback gives referrals to those who still report clinical symptoms). Any individuals who report currently receiving mental health treatment will also be excluded from enrolling in the study and receiving a program. Following rule-out, individuals with a clinical or subthreshold GAD classification will be invited to participate in the study evaluating online interventions. Those who accept will be randomized to one of three conditions: a self-help program (GAD Online), a guided self-help program (Lantern), and a wait-list control group. A complete list of inclusion/exclusion criteria can be found in Textbox 1.

**Inclusion and exclusion criteria for the study.**

Inclusion criteria

- 18+ years old
- Current student at the university
- Meet DSM-V criteria using criterion-based scoring on the GAD-Q-V (clinical) OR score ≥ 5.7 using dimensional scoring of the GAD-Q-IV but not meet DSM-V clinical criteria (subthreshold)
- Provide an email address
- Consent to participate in the study

Exclusion criteria

- Currently receiving mental health treatment
- Current diagnosis of PTSD (PCL-5 score ≥ 38)

#### Ethical Concerns and Consent

The trial protocol has been granted ethical approval by the Institutional Review Board at Stanford School of Medicine (protocol number 31629) and governing bodies at each of the participating colleges. The protocol is registered with ClinicalTrials.gov (NCT02410265).

In the online consent form, students are notified of how their privacy and confidentiality of their data will be maintained. All survey data will be gathered and stored on Qualtrics, an online survey platform with industry-standard data security measures. Students are informed that their participation in the survey or in a program is private and will not be shared with their college, their parents, their peers, etc. Furthermore, all data gathered will be aggregated and deidentified prior to use in any publications. Only the research coordinator has access to identifying student data. Program coaches assigned to work with students in the guided Lantern intervention connect with students via the secure program platform; no personal contact information (eg, email, phone number) is exchanged. As Lantern users receive email notifications when they receive a message from a program coach, Lantern has access to the student users’ emails; however, Lantern is HIPAA compliant and follows appropriate data management and sharing protocols. Students assigned to the GAD Online intervention are provided with generic account user names and passwords, meaning GAD Online never has need for or access to students’ email addresses.

#### Baseline Assessments

After giving online consent, students complete the baseline assessment, which also serves as the screen for eligibility. Table 1 provides a summary of the measures that will be used.

#### Randomization

Randomization will be carried out by the research coordinator after the baseline assessment. Random allocation to the treatment groups will occur within 4 weeks after the screening/baseline survey has been completed. The algorithm for random allocation will consist of a stratified block design, with stratification by level of symptoms (clinical or subthreshold) and a block size of 6. There will be 2 strata, corresponding to clinical/subthreshold symptom level. Allocation will be administered using Randomizer, a Web-based patient randomization service for clinical trials.

#### Risk Management Protocol

Risk management procedures were developed collaboratively with the participating universities, on-campus counselors, online program providers (ie, GAD Online and Lantern), and the research team. All students will be provided with the contact information for the respective on-campus counselor at the start and finish of the online survey. In this way, anyone exposed to the survey will be informed about currently accessible services. Furthermore, all universities have agreed to increase the current time of the existing on-campus counselor should student demand require it. In addition, for any individual in the guided intervention (Lantern), program coaches will monitor for worsening symptoms and express student preference to receive more intensive treatment and provide a referral to the on-campus counselor as necessary. Additionally, if a student in the guided program indicates thoughts of harm to self or others, the coach will notify the coach clinical supervisor (a clinical instructor at Stanford and licensed mental health care professional in the United States) via email and the campus-specific, on-site counselor via phone. Both will help determine how best to manage the situation, and the on-campus counselor will intervene and reach out to the student as necessary. In the consent, students are informed that confidentiality can be breached should the student indicate harm to self or others. Those in the unguided intervention will be explicitly informed that their actions in the program are not monitored and that they should contact the on-campus counselor should they want live support. They will again be reminded of the contact information of the on-campus counselor when they are assigned into this unguided intervention.

#### Sample Size Estimation

The sample size estimation is based on detecting differences in GAD symptom severity change (measured using dimensional scoring of the GAD-Q-IV) from baseline to post- and follow-up assessments between each active conditions and the wait-list control group. Treatment trials of online, CBT-based, guided self-help interventions for GAD have found an average large effect size of roughly 1.07 relative to a wait-list control group using the GAD-Q-IV [38]. In this study, more than half of the treated participants had recovered according to a structured interview, suggesting that a treatment effect size of 1.07 is clinically meaningful. Treatment trials of purely self-help interventions for subthreshold and clinical anxiety have found smaller yet still promising effect sizes ranging from 0.62 to 0.84, respectively, relative to a wait-list control group [31].

To enable detection of difference in mean change between each active intervention and the control group, the sample size is powered to detect at least a medium effect size of 0.5. With 80% power and assuming a 5% significance level, a minimum total sample size per group of 64 is required. Assuming the GAD-Q-IV SD is 3.46 [49], the proposed sample size provides 80% power to detect a between-group effect corresponding to a GAD-Q-IV change of 1.73 points (or a Cohen’s *d* of 0.5 or greater). By targeting 100 students recruited per condition (N=300), even allowing for nearly 30% attrition, which is a possibility in Internet-based intervention trials, GAD-Q-IV mean differences between each active condition compared with the wait-list control condition will be detected with sufficient power. The difference in mean change between the two active conditions is not a primary comparison, as they are two different programs.

### The Interventions

#### Active Condition 1: “Lantern” Guided Self-Help Intervention

**Figure 2:**
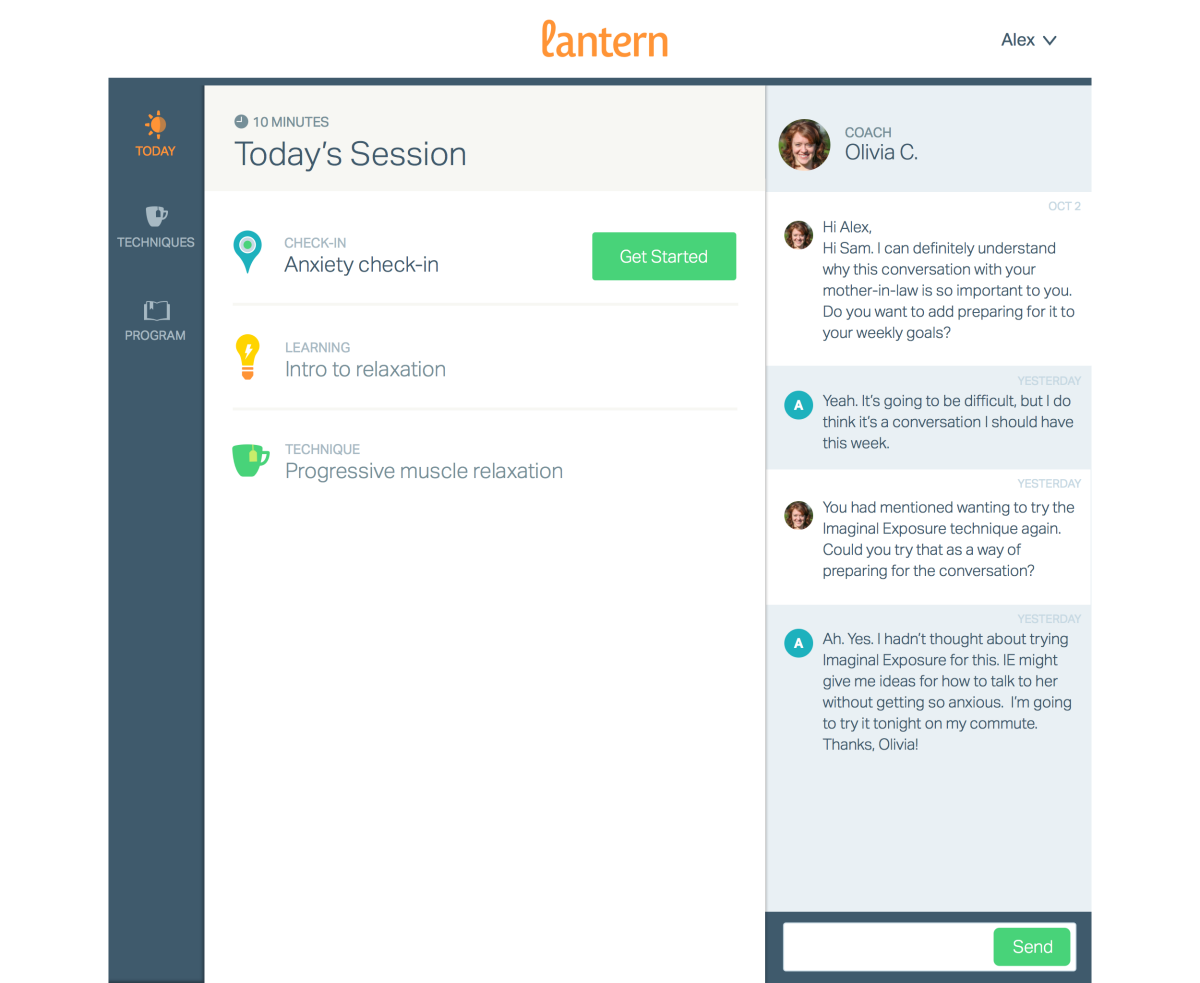
Screenshot of Lantern Internet-based user view.

The Lantern Anxiety Program is a cognitive-behavioral intervention (CBT) that can be accessed via any Internet-enabled computer, mobile phone, or tablet via the Go Lantern website (Figure 2). The program includes psychoeducational content, interactive tools and exercises, symptom monitoring, and an online program coach who can monitor a user’s progress in the program and provide personalized feedback and encouragement via in-program messaging and voice calls. The program is based on an evidence-based, 14-session CBT for GAD intervention, developed by Dr Michelle Newman [36,50,51]. CBT for GAD has been demonstrated to produce the largest effect sizes when compared to other therapy conditions such as analytic psychotherapy and nondirective therapy [52]. The CBT for GAD treatment includes (1) applied relaxation training, which involves the identification of early cues of anxiety, learning the skills of progressive relaxation (PR) and other relaxation techniques and learning how to apply relaxation to anxiety cues (called “applied relaxation,” [AR]); (2) imaginal rehearsal via self-control coping desensitization, using AR and alternative, nonanxious coping thoughts; and (3) cognitive therapy methods to help change how clients perceive, interpret, and believe, so that they will see less threat in the world and feel more confidence in their abilities to cope with the future.

Content is divided into 8 units, each with 5 sessions, resulting in 40 daily 10-minute sessions. The 8 units comprise an introduction to anxiety, automatic thoughts, cognitive reframing, introduction to behavior change, imaginal exposure, situational exposure, mindfulness, and habit formation. There are both cognitive (eg, worry tool, mindfulness) and behavioral (eg, progressive muscle relaxation, deep breathing, guided imagery) techniques taught in each module and always accessible thereafter. Users can access one session per day, with the next session unlocking only after the prior session is completed; therefore, less engaged users may not be exposed to all content. The program and program coach prompt users to schedule reminders to facilitate self-monitoring, encourage completion of scheduled activities, and promote use of tools and techniques in the program. Users can schedule reminders to complete sessions and to use techniques or self-monitoring entries at their desired frequency. They are prompted to do so after learning a new technique. Coaches can also schedule reminders for themselves to send users messages.

Students using Lantern will be guided through the program by mental health workers in India who have been trained to serve as online program coaches. To be eligible to be a coach, individuals must have some background and/or experience in psychology, have at least 6 months of supervised, in-person counseling experience, own a personal computer or smart device and check email daily, be comfortable with technology, and have a minimum of 3 hours per week to dedicate to coaching. A total of 20 potential program coaches were recruited, all of whom had completed a 6-month training course in the basic principles of psychological counseling from the Hyderabad Academy of Psychology based in Hyderabad, India.

A pretraining assessment was used to assess existing skill levels and inform training content development. To begin, 4 weeks of remote training covering basic foundational concepts was conducted. Manuals and papers reviewing CBT for GAD, panic disorder, and social anxiety were assigned, and a virtual discussion session was conducted each week via online collaboration platform GoToMeeting. In addition, coaches were provided a demo user account of the Lantern Anxiety Program so that they could familiarize themselves with the intervention content. Following remote training primarily reviewing content and basic concepts, a 3-day in-person training was conducted in Hyderabad, India, in December 2014. During this training, the Lantern coaching platform was introduced and coaches learned how to effectively interact with the coaching dashboard. The coaching dashboard enables coaches to monitor the activity and engagement of the students assigned to them as well as to send messages to them. Coaches were instructed on guided self-help coaching best practices, introductory phone call and messaging protocols, motivational interviewing, and risk management procedures.

Following training, coaches were assessed for comfort with technology via direct observation of their interaction with the Lantern coaching dashboard during interactive role plays. Coaches who demonstrated low technology fluency and experienced usability issues interacting with the coaching software in comparison with the group were asked to attend additional training and practice sessions to continue. Behavioral rehearsal tasks were used to evaluate coaches’ fidelity to the coaching protocol. Coaches were required to complete a mock introductory phone call with a control student (played by the research coordinator or volunteer students following a script), and fidelity to the phone call procedural checklist was assessed. Coaches were also required to respond to message prompts designed to assess their mastery of core competencies of CBT for GAD in the context of a guided self-help intervention. Their responses were rated against a messaging best practices checklist that was developed by the authors. Finally, coaches had to complete an established “e-therapist assessment” developed by the National eTherapy Centre (NeTC) at Swinburne University of Technology [53] and score at least 80%. Of the 20 trainees who began training, 65% (n=13) cleared the post-training evaluation and moved forward to become program coaches for this trial.

Throughout the intervention period, coaches will have on-going monitoring and support. Coaches must attend a weekly, live 1-hour supervision, during which coaches can present any challenging cases to the group and receive feedback from the coach clinical supervisor (a clinical instructor at Stanford and a licensed mental health care professional in the United States). Coaches will also be asked to submit 1 challenging message exchange per week to a clinical psychology doctoral student for individual feedback on messaging style and content. Finally, coaches can post questions and messages on a group listserv to get feedback from the supervisor as well as from peers.

#### Active Condition 2: “GAD Online” Pure Self-Help Intervention

**Figure 3:**
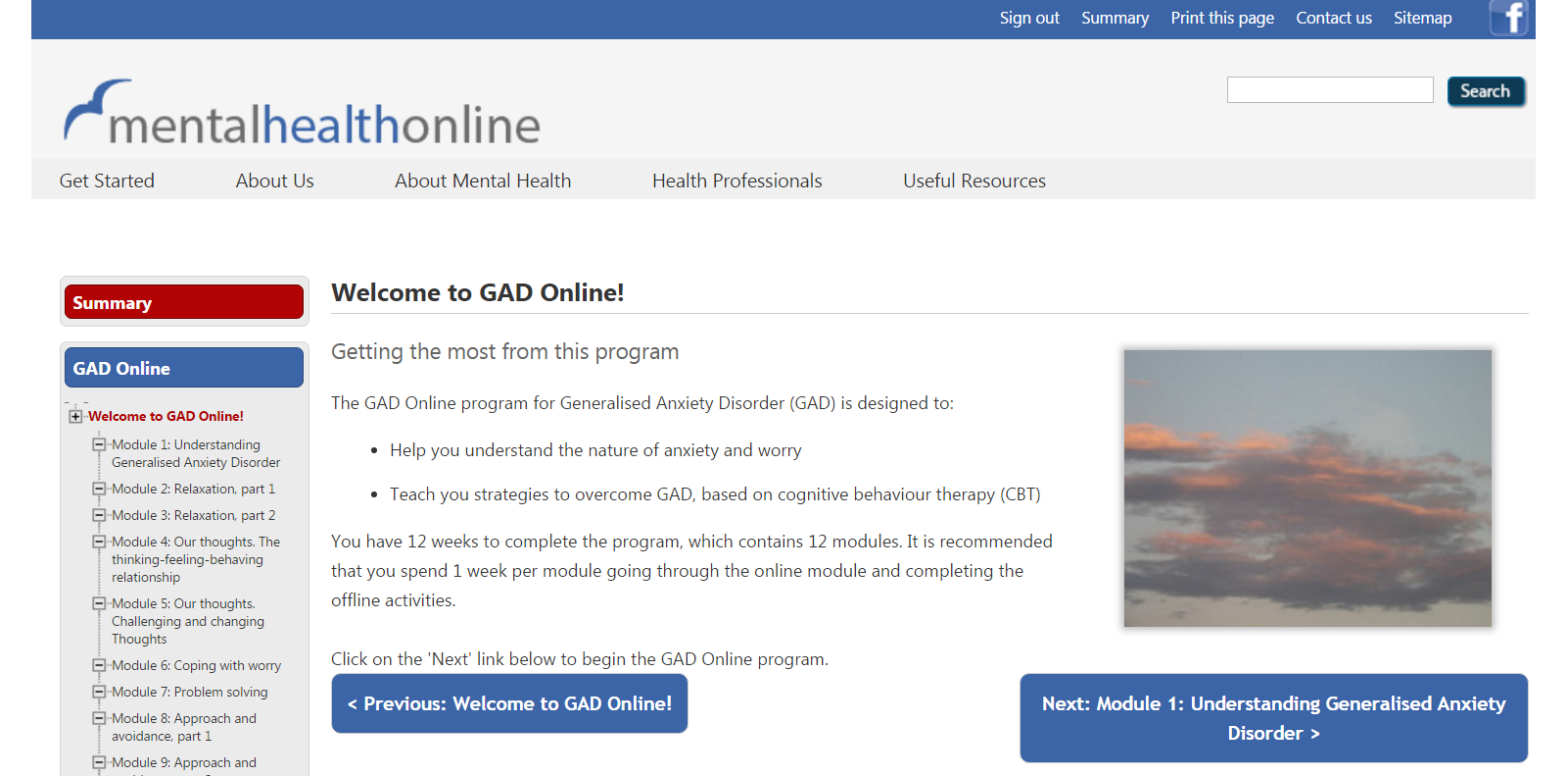
Screenshot of Mental Health Online’s GAD Online program.

The GAD Online program is also CBT based and can be accessed via any Internet-enabled computer, mobile phone, or tablet via the Mental Health Online website (Figure 3). Mental Health Online is a suite of online mental health programs developed and maintained by the NeTC at Swinburne University of Technology and funded by the Australian Federal Government Department of Health. The GAD Online program delivered in this trial, which was previously reported on as an Anxiety Online module [33], provides psychoeducation about GAD and techniques to reduce GAD symptoms (eg, relaxation, challenging thoughts, coping with worry, problem solving). The content covered in this program is comparable to that delivered in the Lantern GSH program. Content is divided into 12 modules, with 1 module per week being the suggested use; however, users can progress at their own pace. Unlike the design of the Lantern program, users can access other modules in the GAD Online program even if they do not complete the first module. Students are explicitly told they are not monitored and should contact the on-campus counselor should they require real-time support from a professional.

#### Wait-list Control Condition: Delayed Program

Students randomized to the wait-list control condition will complete surveys at the postintervention time point (3 months) and the follow-up time point (9 months). Following successful completion of these surveys, they will be allocated to one of the online interventions. Students will be assessed for whether they sought help from the on-campus counselor at each time point. The counselor’s contact information is available to all students across the campus.

### Minimization of Contamination

As randomization occurs within each college campus, there is a possibility of contamination, in that students could share their programs with each other. However, this type of contamination is not believed to be likely, as the programs are presented as personalized and private. Furthermore, because recruitment for this exploratory trial is occurring at 3 distinct colleges, selected more so due to partnership, comparability between sites is not known. Future, larger trials might consider randomization by college site.

### Measures

Outcome data will be collected at post-treatment (3 months) and 6-months follow-up (9 months). The 3-month outcome is the primary end point as the intervention delivery will be completed and the optimal effect of the treatment would be expected. The 6-month follow-up is included to evaluate the sustainability of the effect of the intervention. The outcome assessment measures are summarized in Table 1. Although the primary outcome measure is the GAD-Q-IV, we also assessed other anxiety disorders and potential functional impairments. Because only a few studies have gathered comprehensive data on prevalence rates of types of anxiety disorders and given the unique opportunity to administer an online survey to this large population, a subaim of this study and the involved stakeholders is to gather epidemiological data that can inform future research and policy and programmatic decisions by stakeholders to address the needs of students. Besides, given the high degree of comorbidity present among individuals who experience GAD [6,36,54], measuring psychological state and evaluating co-occurring disorders such as other anxiety disorders and depression are important. Finally, because CBT for GAD has been demonstrated to address symptoms associated with other disorders [47,55], both the prevalence and secondary outcomes are relevant. To ease anticipated survey burden, the survey is designed to use logic that allows for participants with low scores and fewer comorbid concerns to “skip” subsections. Demographic data will include gender, age, race/ethnicity, sexual orientation, religion, relationship status, family income, and hometown.

### Details of the Measurements

#### Generalized Anxiety Disorder Questionnaire (4th Edition)

The primary outcome is GAD symptom severity as measured by dimensional scoring of the 4th edition of the Generalized Anxiety Disorder Questionnaire (GAD-Q-IV) [42]. GAD caseness, as measured by categorical scoring of the GAD-Q-IV, is a secondary measure. The GAD-Q-IV is a 9-item self-report measure designed as an initial screen for the presence of GAD based on the DSM-IV. The GAD-Q-IV showed 89% specificity and 83% sensitivity when compared to structured interview diagnoses of individuals with GAD, social phobia, panic disorder, and a nonanxious comparison group. The GAD-Q-IV has demonstrated good test-retest reliability in a college sample over a 2-week assessment, with 92% of the sample showing stability across time with respect to GAD diagnosis [42].

#### Emotional Distress From Anxiety Measure

The Patient Reported Outcomes Measure Information System (PROMIS) Emotional Distress from Anxiety measure [56] is a reliable 8-item self-report measure to evaluate emotional distress from anxiety. This measure was included as a potential global outcome measure of improved quality of life.

#### Penn State Worry Questionnaire

The Penn State Worry Questionnaire (PSWQ) [57] is a 16-item self-report measure of the frequency and intensity of worry. The PSWQ has been shown to distinguish individuals with GAD from individuals with other anxiety disorders [58]. This measure was included to assess change in worry, a primary symptom of GAD.

#### Panic Disorder Self-Report

The Panic Disorder Self Report (PDSR) [44] is a 22-item self-report measure designed to diagnose panic disorder based on DSM-IV criteria. The PDSR showed 100% specificity and 89% sensitivity when compared with clinician-based ADIS-IV-L diagnoses of individuals diagnosed with panic disorder, GAD, social phobia, and a nonanxious comparison group. This measure, along with the other measures of anxiety disorders and depression, was included to assess both prevalence of this type of anxiety and the impact of the active interventions on symptoms associated with typically co-occurring disorders.

#### Social Phobia Diagnostic Questionnaire

The Social Phobia Diagnostic Questionnaire (SPDQ) [43] is a 10-item self-report measure designed to diagnose social phobia based on DSM-IV criteria. The SPDQ showed a specificity of 82% and a sensitivity of 85% when compared with clinician-based structured interview diagnoses of individuals meeting criteria for social phobia, panic disorder, and a nonanxious comparison group.

#### Post-Traumatic Stress Disorder Checklist

The Post-Traumatic Stress Disorder Checklist for DSM-V (PCL-5) [45] is a 20-item self-report measure that assesses the 20 DSM-V symptoms of PTSD. It was designed to monitor symptom change during and after treatment, screen individuals for PTSD, and make provisional PTSD diagnoses.

**Table 1:** Scales to be administered at each time point.

Outcome variable			Measurement tool/data collection method	Time point (months)		
				0	3	9
Eligibility			Self-report	X		
Demographics			Self-report	X		
Field of study			Self-report	X		
Perceived social support			Self-report	X		
Belief in efficacy of online programs (credibility/expectancy)			Self-report	X		
Contamination			Self-report		X	
Grade point average			Self-report	X	X	X
**Primary outcome**						
	Difference in GAD symptom severity at postintervention and follow-up between the intervention groups and control group		4th edition of the Generalized Anxiety Disorder Questionnaire (GAD-Q-IV) (scored dimensionally)	X	X	X
**Secondary outcomes**						
	**Anxiety**					
		GAD caseness	GAD-Q-IV (scored categorically)	X	X	X
		Emotional distress from anxiety	PROMIS Emotional Distress from Anxiety measure	X	X	X
		Worry	Penn State Worry Questionnaire (PSWQ)	X	X	X
		Panic disorder symptoms	Panic Disorder Self-Report (PDSR)	X	X	X
		Social anxiety symptoms	Social Phobia Diagnostic Questionnaire (SPDQ)	X	X	X
		PTSD symptoms	Post-Traumatic Stress Disorder Checklist for DSM-V (PCL5)	X	X	X
		OCD symptoms	LEVEL 2—Repetitive Thoughts and Behaviors—Adult (adapted from the Florida Obsessive-Compulsive Inventory [FOCI] Severity Scale [Part B])	X	X	X
		Distress severity for specific fears	Self-report	X	X	X
	**Depression**					
		Depression symptoms	Depression Anxiety Stress Scales (DASS21)	X	X	X
	**Behavioral measures**					
		Alcohol consumption	Self-report	X	X	X
		Help seeking	Self-report	X	X	X
		Medications	Self-report	X	X	X
	**Functional measures**					
		Sleep	Insomnia Severity Index (ISI)	X	X	X
		Difficulties with emotions and relationships	Strengths and Difficulties Questionnaire (SDQ)	X	X	X
		Satisfaction with social roles	PROMIS Satisfaction with Social Roles and Activities measure	X	X	X
		Nonspecific distress	Kessler Distress Measure (K10)	X	X	X
		Confidence in ability to manage anxiety symptoms	General Perceived Self-efficacy Scale (GSE)	X	X	X
		Motivation to work on anxiety	Self-report	X	X	X
	**Process measures**					
		Program use and engagement	Self-report		X	
		Usefulness of GAD Online or Lantern	Self-report		X	
		Satisfaction with GAD Online or Lantern	Client Satisfaction Questionnaire (CSQ)		X	

#### Repetitive Thoughts and Behaviors

The LEVEL 2—Repetitive Thoughts and Behaviors—Adult (adapted from the Florida Obsessive-Compulsive Inventory [FOCI] Severity Scale [Part B]) [59] assesses obsessive-compulsive disorder symptoms. This emerging measure of the DSM-V was developed to be administered at the initial patient interview and to monitor treatment progress.

#### Depression Anxiety Stress Scales-Short Form

The Depression Anxiety Stress Scales-Short Form (DASS21) [60] is a 21-item self-report measure designed to measure negative emotional states of depression, anxiety, and tension or stress. The DASS is divided into 3 self-report scales. The depression scale assesses dysphoria, hopelessness, devaluation of life, self-deprecation, lack of interest/involvement, anhedonia, and inertia. The Anxiety Scale assesses autonomic arousal, skeletal muscle effects, situational anxiety, and subjective experience of anxious affect. The Stress Scale is sensitive to levels of chronic nonspecific arousal, assessing difficulty relaxing, nervous arousal, and being easily upset/agitated, irritable/over-reactive, and impatient. All scales have been shown to have high internal consistency and to yield meaningful discriminations in a variety of settings.

#### Insomnia Severity Index

The Insomnia Severity Index is a 7-item self-report questionnaire to assess insomnia severity [61]. It has adequate internal consistency and is a reliable self-report measure to evaluate perceived sleep difficulties. This measure, along with the other measures of general functioning, was included to assess the impact of the active interventions on functional impairments typically associated with GAD.

#### Strengths and Difficulties Questionnaire

The Strengths and Difficulties Questionnaire [62] assesses self-reported difficulties with emotions, concentration, and relationships. It is a reliable short-form, self-report assessment of inattention, peer relationships, and pro-social behavior, factors that, if improved, could indicate positive impact of the active interventions.

#### Satisfaction With Social Roles

The PROMIS Satisfaction with Social Roles and Activities measure [63] is an 8-item self-report assessment of satisfaction with one’s ability to perform daily activities and meet the needs of various relationships.

#### Kessler Distress Measure

The Kessler Distress Measure [64] assesses nonspecific distress. The 10-question (K10) self-report scale has good precision as well as consistent psychometric properties across major sociodemographic subsamples. This measure was included as an assessment of a global outcome of reduced general distress.

#### General Perceived Self-Efficacy Scale

The General Perceived Self-Efficacy scale (GSE) [65] is a 10-item self-report measure that assesses beliefs in one’s capability to handle new and difficult tasks and adaptive challenges after experiencing stressful life events. This measure was included because enhanced self-efficacy is considered one of the main psychological benefits of self-help interventions [66].

### Program Use and Engagement

Program usage will be examined for the 2 active intervention groups with respect to 3 indices: frequency of logins, frequency of self-monitoring, and number of modules accessed. Reason for drop out will be assessed via self-report questions in the survey as well as a semistructured interview with those who are willing to enroll in a follow-up substudy. For both the GAD Online and Lantern platforms, module access can be passively monitored. Engagement in Lantern can be further qualified in terms of engagement with the program coach. The Lantern software can program reminders for coaches to reach out to inactive users after a specific period. Coaches are given training in motivational interviewing and encouraged to use these skills to help engage users and address fluctuating stages of change during the intervention. Unstructured data (ie, text) from users’ program entries and correspondence with coaches can be qualitatively (eg, language used) and quantitatively (eg, user: coach message frequency) evaluated, with participant consent and understanding that message content can be used for research, to examine motivational strategies and engagement.

### Program Satisfaction

Program satisfaction will be examined for the 2 active intervention groups using the Client Satisfaction Questionnaire (CSQ) [67]. The CSQ is an 8-item self-report statement of satisfaction with health and human services. The generic questionnaire has been customized for each intervention (eg, replacing the term “service” with “the GAD Online program” or “the Lantern Anxiety Program”). Those using Lantern will also provide feedback on their experience working with the program coach.

### Data Management

All data will be stored on secure servers hosted by Qualtrics, and data downloaded from those servers will be managed and stored on encrypted computers accessed only by members of the Stanford research team.

### Analysis

#### Descriptive Analyses

Chi-squares (categorical variables) and *t* tests (continuous variables) will be used to compare demographic variables and baseline scores on the outcome measures for the 3 groups. Findings will be reported according to the CONSORT guidelines [68], including a trial flowchart. This will include total students assessed for inclusion and exclusion criteria within period of screening, number of students meeting inclusion or exclusion criteria, number screened for eligibility, number agreeing to enter the intervention trial, and number refusing or excluded (with reasons). The number continuing through the trial, actively withdrawing, and passively lost to follow-up will be shown by arm. The outcome measures will be summarized at baseline/screening, at 3-month and 9-month follow-up by the intervention arm and overall.

#### Outcome Analyses

A 3 condition × time mixed-design ANOVA will be conducted to examine both primary and secondary hypotheses. Using a mixed-design ANOVA will test for differences between 2 or more independent groups while subjecting participants to repeated measures. The model is a type of mixed effect model, with the fixed effects factor being a between-subjects variable and the random effects factor being a within-subjects variable. Follow-up paired comparisons will be conducted to specifically assess if there is significant differential change in GAD symptoms between each active condition and control from the baseline to the postcondition and follow-up assessments. Within- and between-group effect sizes will be calculated using Cohen’s *d* (based on the pooled standard deviation), specifically contrasting each active condition with the control condition for the main outcome.

Primary analyses will be undertaken using an intention-to-treat (ITT) approach, including all participants randomized regardless of treatment actually received, program engagement, or withdrawal from the trial. Using a mixed model design will allow us to include participants with missing data, which is likely to occur in large Internet-based trials.

Study site will be included as a covariate as this might have an influential effect given that the 3 sites were selected primarily based on accessibility versus between-site comparability. If some sites have poor recruitment, the data will be combined.

#### Mediator and Moderator Analyses

Potential mediators and moderators of the efficacy of the intervention will also be explored. For example, level of program engagement, defined by number of sessions completed and number of days logged on to the program, will be evaluated for mediation of outcomes.

Additionally, given that randomization was stratified between clinical and subthreshold populations, whether symptom severity influences the efficacy of the intervention to reduce GAD symptoms can be explored. Additional predictors of outcome that will be explored include medication use, motivation to work on anxiety, belief in efficacy of online programs, perceived social support, and grade point average. Medication use will be controlled for in the analyses if the sample is large enough; otherwise, the results will be provided qualitatively.

## Results

The study commenced in February 2015. The sample was recruited over a 3-week period at each college. The trial is expected to end in December 2015.

## Discussion

### Potential Impact

This trial represents an opportunity to explore whether technology can be leveraged to reduce the significant mental health treatment gap in India, particularly for students in Indian universities. This will be the first trial to examine the feasibility, acceptability, and efficacy of Internet-based, unguided and guided self-help interventions to reduce GAD symptoms in Indian university students. It will also be the first trial evaluating the acceptability and feasibility of online, guided self-help interventions supported by trained mental health workers in India.

Intervening to reduce GAD symptoms in students is important because increasing GAD symptomatology is associated with greater functional impairment [11], reduced quality of life [12], greater disability and help seeking [13,14], and greater costs of health care [15]. Furthermore, without treatment, GAD symptoms have a chronic course and persistent symptoms [9]. Given the increasing prevalence of Internet connectivity and mobile phone penetration [69] and in the face of limited mental health care professional availability, delivering evidence-based mental health care via the Internet may be particularly attractive to individuals in developing countries, particularly students in Indian universities, who have limited access to mental health care services. Furthermore, both programs that will be employed, GAD Online and Lantern, are scalable platforms, thus making the use of Internet-based, unguided and guided self-help interventions a practical approach. If the interventions are found to be effective, these programs can be more specifically adapted for this population and then be promoted and disseminated seamlessly to the larger student population, universities, and younger individuals.

### Limitations

Relying entirely on self-report data to assess individuals is a limitation of this study and implies that the allocation of interventions may not be as reliable as if diagnostic interviews and other objective measures were used. This method was selected for two reasons. First, one of the primary barriers to help seeking is stigma, particularly in this population. In stakeholder discussions, both administrators and students highlighted that concerns around confidentiality and disclosure to others – peers, professors, parents – prevent many students from visiting the existing on-campus counselor. Thus, a private, entirely online approach is likely to achieve greater participation and more accurate responses. Second, one of the main goals of this model of intervention is to develop a cost-effective and sustainable way to disseminate and evaluate these programs at scale, and requiring an in-person assessment would dramatically increase costs and reduce sustainability. Moreover, these assessments are meant to inform recommendations about program "matches" that meet participants' self-reported needs, and participants are empowered to choose the avenue of support they perceive to be the best fit (ie, on-campus counselor or online program). Following initial trials establishing feasibility, clinical interviews can be conducted along with self-report assessments to evaluate the validity and reliability of the self-report screening measures for this population. Additionally, in future trials, the real and important limitation of response burden could be addressed and fewer, more targeted measures could be administered.

Another limitation is that the primary outcome measurement tool, the GAD-Q-IV, and the associated diagnostic thresholds have been normed in US university student populations but not in an Indian university student population. One implication is that the prevalence of the disorder could be mischaracterized due to diagnostic criteria being interpreted differently cross-culturally. For example, the DSM-IV GAD criterion that anxiety be “excessive” might lead to underdiagnosis of GAD in developing countries in which worry is less likely to be reported excessive in the presence of comparatively more severe life concerns [70]. Using dimensional versus categorical scoring of the GAD-Q-IV to both determine eligibility and monitor for change in symptomatology, this concern might be partially addressed. The cutoff point of 5.7 was found to be overly sensitive in the US college population [42], so it is a reasonable threshold above which to initially allocate interventions to those potentially in need but not currently accessing services. However, future and parallel research should focus on validating the assessment tools.

Another potential limitation is the fact that the two active conditions differ both by the degree of coaching support and the program used, which precludes this study from clearly deducing the additional, if any, benefit of guidance. However, the primary hypothesis is that the use of one or both of the active interventions will lead to a greater GAD symptom reduction than the wait-list control. If the unguided program is found to be both feasible to disseminate to this population and relatively effective, future controlled studies can evaluate the comparative benefit of guidance and conduct cost-benefit analyses of delivering unguided versus guided programs. The conditions under which it is more or less effective will also be explored (eg, it might be more effective for those with higher reported motivation who are more likely to complete more of the program). The primary initial aim is an evaluation of the feasibility and efficacy of the model of survey-linked-to-online-interventions to reduce the incidence and prevalence of common mental health disorders in defined Indian university populations**.**

## Abbreviations

CBT: cognitive behavior therapy
DASS: Depression Anxiety Stress Scales
DSM: Diagnostic and Statistical Manual of Mental Disorders
FOCI: Florida Obsessive-Compulsive Inventory
GAD: generalized anxiety disorder
GAD-Q-IV: 4th edition of the Generalized Anxiety Disorder Questionnaire
GSES: General Self-Efficacy Scale
ISI: Insomnia Severity Index
K10: Kessler distress measure
PCL-5: Post-Traumatic Stress Disorder Checklist for DSM-V
PDSR: Panic Disorder Self Report
PROMIS: Patient Reported Outcomes Measure Information System
PSWQ: Penn State Worry Questionnaire
RCT: randomized controlled trial
SDQ: Strengths and Difficulties Questionnaire
SPDQ: Social Phobia Diagnostic Questionnaire
